# Sparing of normal tissues with volumetric arc radiation therapy for glioblastoma: single institution clinical experience

**DOI:** 10.1186/s13014-017-0810-3

**Published:** 2017-05-02

**Authors:** Tina Marie Briere, Mary Frances McAleer, Lawrence B. Levy, James N. Yang

**Affiliations:** 1 Departments of Radiation Physics, UT MD Anderson Cancer Center, 1400 Pressler St., Unit #1420, Houston, TX 77030 USA; 2 Departments of Radiation Oncology, UT MD Anderson Cancer Center, Houston, TX USA

**Keywords:** VMAT, IMRT, Glioblastoma multiforme, Radiation therapy

## Abstract

**Background:**

Patients with glioblastoma multiforme (GBM) require radiotherapy as part of definitive management. Our institution has adopted the use of volumetric arc therapy (VMAT) due to superior sparing of the adjacent organs at risk (OARs) compared to intensity modulated radiation therapy (IMRT). Here we report our clinical experience by analyzing target coverage and sparing of OARs for 90 clinical treatment plans.

**Methods:**

VMAT and IMRT patient cohorts comprising 45 patients each were included in this study. For all patients, the planning target volume (PTV) received 50 Gy in 30 fractions, and the simultaneous integrated boost PTV received 60 Gy. The characteristics of the two patient cohorts were examined for similarity. The doses to target volumes and OARs, including brain, brainstem, hippocampi, optic nerves, eyes, and cochleae were then compared using statistical analysis. Target coverage and normal tissue sparing for six patients with both clinical IMRT and VMAT plans were analyzed.

**Results:**

PTV coverage of at least 95% was achieved for all plans, and the median mean dose to the boost PTV differed by only 0.1 Gy between the IMRT and VMAT plans. Superior sparing of the brainstem was found with VMAT, with a median difference in mean dose being 9.4 Gy. The ipsilateral cochlear mean dose was lower by 19.7 Gy, and the contralateral cochlea was lower by 9.5 Gy. The total treatment time was reduced by 5 min. The difference in the ipsilateral hippocampal D_100%_ was 12 Gy, though this is not statistically significant (*P* = 0.03).

**Conclusions:**

VMAT for GBM patients can provide similar target coverage, superior sparing of the brainstem and cochleae, and be delivered in a shorter period of time compared with IMRT. The shorter treatment time may improve clinical efficiency and the quality of the treatment experience. Based on institutional clinical experience, use of VMAT for the treatment of GBMs appears to offer no inferiority in comparison to IMRT and may offer distinct advantages, especially for patients who may require re-irradiation.

## Background

The definitive treatment of glioblastoma multiforme (GBM) typically includes maximal safe resection followed by chemotherapy and radiation therapy (RT) [1, 2]. These tumors have inherent radioresistance [3], tend to recur locally [4–9] and recent studies have shown the median time to progression to be 7–9 months [6–9]. Typical non-hematologic acute toxicities associated with chemoradiation can include fatigue, dermatitis, alopecia, dizziness, headache and nausea or vomiting [10, 11]. Patients report lower health-related and functional quality of life than controls [12]. Late toxicity can include persistent fatigue, hearing or vision impairment, neuroendocrine dysfunction, and cognitive deficits [3, 10, 13]. Because life expectancy can be drastically reduced in patients with GBM, maintaining neurological function and the ability to perform daily activities can be an important treatment goal [13–15]. The dose constraints of the adjacent critical normal structures often limit the dose able to be delivered to the tumor target.

Minimizing RT dose to normal tissues is of utmost importance to minimize both acute and late toxicity of treatment. Intensity modulated radiation therapy (IMRT) has become a modality of choice because treatment plans show similar or better target coverage and sparing of normal tissues as compared to 3-dimensional conformal radiation therapy [14, 16–22]. Noncoplanar beams can provide more freedom in the beam arrangement to achieve good conformity, avoid critical structures, and enable fast dose fall-off outside the target. Volumetric arc radiation therapy (VMAT) delivers a modulated beam in one or more arcs, and, in general, VMAT can provide similar target coverage and normal tissue sparing as IMRT while substantially decreasing the treatment time. Treatment planning studies with limited numbers of patients have demonstrated the non-inferiority of VMAT for gliomas compared with IMRT and tomotherapy [20, 22–25].

The use of VMAT for GBMs was adopted at our institution in 2014 due to improvement in the sparing of the brainstem and cochleae without loss of target coverage. The goal of the present work is to present our institutional experience with both treatment modalities. In order to demonstrate the differences in plan quality between VMAT and IMRT treatment plans for glioblastoma, we have analyzed the dose distributions of 90 clinical treatment plans, 45 patients treated with VMAT and 45 with IMRT. Unlike more limited treatment planning studies, all 90 plans were created by clinical dosimetrists and used for patient treatment. For six patients in this study, clinical VMAT and IMRT plans were available, and differences in these plans are also presented to demonstrate the differences in these modalities for this type of treatment.

## Methods

Ninety consecutive adult patients treated at our institution between 2014 and 2015 were included in this retrospective study, 45 treated with step-and-shoot IMRT and 45 with VMAT. Institutional review board approval was obtained for this retrospective analysis. Patients were included if they were diagnosed with GBM, astrocytoma or oligodendroglioma treated using the glioblastoma treatment protocol described below.

Patients were simulated in a supine position, and the head was immobilized in neutral position with a customized thermoplastic mask. Computed tomography images with a slice thickness of 2–3 mm were acquired for treatment planning, and these images were co-registered with each subject’s corresponding brain magnetic resonance images (MRI) to facilitate target and normal tissue delineation. The use of MR imaging has been shown to improve target delineation [26]. The gross tumor volume (GTV) was defined as the volume corresponding to the resection cavity and gadolinium contrast enhancing T1-weighted MRI images. The clinical target volume (CTV) included a 2-cm isotropic expansion from the GTV, modified to include areas of FLAIR abnormalities on the fused MRI compatible with tumor infiltration and to respect normal anatomic barriers. The CTV and GTV were each expanded by 0.3–0.5 cm to create the planning target volume (PTV) and boost planning target volume (bPTV), respectively. The method for defining the GTV and CTV is consistent with ESTRO-ACROP current guidelines [27], but it should be noted that our institution uses a boost PTV in addition to a conventional CTV-derived PTV [28]. The optic chiasm, optic nerves and cochleae were delineated on the simulation CT assisted by the fused T2-weighted MR images. The hippocampi were delineated following RTOG 0933 guidelines [29] and for 84 patients, these delineations were post hoc. The brain contour excluded the GTV, optic chiasm, and brainstem.

All step-and-shoot IMRT plans utilized 5 or 6 noncoplanar beams with 4 couch angles, while VMAT plans used either 2 full or partial coplanar arcs either alone or with an additional partial noncoplanar arc. Both the VMAT and IMRT plans were optimized using the Pinnacle^3^ treatment planning system (Philips HealthCare, Fitchburg, WI). The PTV was prescribed a dose of 50 Gy in 30 fractions, while the bPTV was prescribed 60 Gy, to be delivered using a simultaneous integrated boost technique. The following dose constraints were used during treatment planning: the maximum point dose (D_max_) to the optic chiasm and optic nerves was ≤ 54 Gy, for each eye D_max_ ≤ 40 Gy and the mean dose (D_mean_) was ≤ 30 Gy, for each cochlea D_max_ ≤ 45 Gy and D_mean_ ≤ 30 Gy, the volume of brain receiving ≥ 30 Gy (V_30Gy_) was ≤ 50%, and for the brainstem V_30Gy_ ≤ 33% and the V_60Gy_ was ≤ 0.01 cc. The hippocampi were not considered as avoidance structures, and therefore no dose constraints were applied during treatment planning. Patients were treated on Varian 2100EX, Trilogy or TrueBeam linear accelerators with either Millennium or high definition multileaf collimators (Varian Medical Systems, Palo Alto, CA). One patient was treated on an Elekta VersaHD linear accelerator (Elekta, Stockholm, Sweden). The total treatment time from the start of imaging to the final beam-off time was calculated from the Mosaiq Record and Verify system (Version 2.6, IMPAC Medical Systems, Sunnyvale, CA).

Statistical analysis of categorical variables such as tumor location was performed using Fisher’s exact test, while continuous variables such as dose and treatment volume were compared using the Wilcoxon-Mann–Whitney rank sum test. Statistical significance was adjusted using the Bonferroni correction, with *P* = 0.05/10 = 0.005 for the target volumes and *P* = 0.05/36 = 0.0014 for the OARs. Relevant minimum, mean, maximum and percentage dose volume histogram variables were studied. In addition, the gradient index (GI) at the 50 Gy and 60 Gy levels was calculated for the total brain using the equation [30]:

$$ G{I}_D=\frac{Vo{ l}_{0.5 D}}{Vo{ l}_D} $$

where a smaller value indicates a steeper falloff between, for example, the 50 Gy and 25 Gy isodose lines.

Finally, the inhomogeneity index (II) was calculated for both PTVs using the following equation [20]:

$$ I I={D}_{05\%}-{D}_{95\%} $$

where D_XX%_ is the dose that covers XX% of the target volume, and a higher value indicates a less homogeneous dose distribution. The ideal value of II for the boost PTV would be 0 Gy and for the PTV it would be ≤ 10 Gy. Previous studies have shown improved sparing of organs at risk (OARs) with high definition multileaf collimators [31], and so a separate analysis of the PTV and select OARs was performed for the high definition plans.

Six patients were planned using both IMRT and VMAT. For these patients, the VMAT plans were chosen for clinical treatment, and were also included in the larger VMAT cohort analyzed in this work. Comparison of target coverage and dose to critical structures using these subjects was made to illustrate the differences between these two modalities. Statistical analysis was performed using the Wilcoxon signed-rank test. However, it should be noted that the minimum possible *p*-value for 6 data points exceeds the values for significance after Bonferroni adjustment.

## Results

Characteristics of the patients and treatment techniques are shown in Table 1. For the two different patients cohorts (IMRT vs. VMAT), there was no statistical difference in diagnosis, tumor location, tumor side, planning target volumes and brain volume between the treatment types. The required number of monitor units was greater for the VMAT plans, but the median total treatment time of the VMAT plans was 5 min or 50% shorter than for the IMRT plans.

**Table 1:** Characteristics of the 90-patient cohort

Characteristic	Number of patients/Median value (Range)		P*
	IMRT	VMAT	
Diagnosis			0.49
Glioblastoma	44	43	
Oligodendroglioma	1	0	
Astrocytoma	0	2	
Tumor Location			0.32
Frontal	14	23	
Occipital	2	1	
Parietal	8	6	
Temporal	13	6	
Thalamus	1	1	
2 or more lobes	7	8	
Tumor Side			0.34
Bilateral	4	5	
Left	24	17	
Right	17	23	
bPTV Volume (cc)	85.1 (11.0–328.5)	81.4 (28.3–285.3)	0.94
PTV Volume (cc)	359.3 (128.9–779.1)	370.8 (193.9–877.5)	0.18
Brain Volume (cc)	1301.5 (1022.2–1849.0)	1326.9 (1016.0–1658.6)	0.23
# Beams			<0.001
2	0	38	
3	0	7	
5	43	0	
6	2	0	
# Couch Angles			<0.001
1	0	39	
2	0	6	
4	45	0	
Multileaf Collimator			0.02
High Definition	33	42	
Standard	12	3	
# Monitor Units	347 (285–487)	453 (274–665)	<0.001
Treatment Time	15.0 (7.7–21)	10.0 (6.3–15.3)	<0.001

*IMRT* intensity modulated radiation therapy, *VMAT* volumetric arc therapy, bPTV boost planning target volume prescribed to 60 Gy, PTV planning target volume prescribed to 50 Gy

*From Fisher’s exact test or Wilcoxon-Mann–Whitney rank sum analysis

Table 2 lists the dose delivered to the targets by either the IMRT or VMAT plans. Differences in the bPTV doses were not statistically significant. The median bPTV D_mean_ differed by 0.1 Gy between the IMRT and VMAT cohorts, while the median D_max_ was slightly lower and V_60Gy_ was slightly higher for the VMAT cohort. With both IMRT and VMAT, the prescription isodose line covered at least 90% of the bPTV for all patients. For the PTV, D_mean_ was lower by 0.5 Gy (*P* < 0.001) for the VMAT cohort. Differences in D_min_, D_max_ and V_50Gy_ coverage were not statistically significant. At least 95% of the PTV was covered by the prescription isodose line with both treatment approaches. Finally, the II for the bPTV and PTV were not significantly different between IMRT and VMAT.

**Table 2:** Planning target volumes for the 90-patient cohort

	Median (Range)		
	IMRT	VMAT	P*
bPTV			
D_min_ (Gy)	56.8 (48.4–59.8)	58.2 (49.4–60.0)	0.15
D_mean_ (Gy)	61.8 (61.1–62.5)	61.9 (61.1–62.8)	0.02
D_max_ (Gy)	64.1 (62.6–66.4)	63.8 (62.0–65.5)	0.05
V_60Gy_ (%)	99.0 (90.5–100.0)	99.4 (94.7–100.0)	0.19
II (Gy)	2.2 (1.2–5.3)	1.9 (0.8–3.1)	0.04
PTV			
D_min_(Gy)	45.3 (33.6–48.7)	42.7 (23.9–48.1)	0.003
D_mean_ (Gy)	56.9 (53.8–58.7)	56.4 (54.2–59.3)	<0.001
D_max_ (Gy)	64.1 (62.6–66.7)	63.8 (62.0–65.5)	0.05
V_50Gy_ (%)	99.1 (95.1–99.9)	98.5 (95.2–99.9)	0.02
II (Gy)	11.2 (9.8–12.7)	11.6 (10.2–12.5)	0.07

*IMRT* intensity modulated radiation therapy, *VMAT* volumetric arc therapy, *bPTV* boost planning target volume prescribed to 60 Gy, *PTV* planning target volume prescribed to 50 Gy, *D*_*min*_ minimum dose, *D*_*mean*_ mean dose, *D*_*max*_ maximum dose, *V*_*XXGy*_ volume receiving XX Gy or more, *II* inhomogeneity index

*From Wilcoxon-Mann–Whitney rank sum analysis

With respect to the OARs (Table 3), the differences in brain D_mean_, D_max_, V_30Gy_ or V_60Gy_ were not statistically significant between IMRT and VMAT. The GI within the brain was not statistically significant at 50 Gy or 60 Gy. The differences in dose to the optic chiasm, optic nerves, hippocampi, eyes and lenses were also not statistically significant. The difference in the ipsilateral hippocampal D_100%_ was 12 Gy, though with *P* = 0.03 this is not statistically significant. There was a large difference in brainstem D_mean_, with the median value for the VMAT cohort being 9.4 Gy lower than the IMRT cohort (*P* < 0.001). The median D_mean_ to the ipsilateral cochlea was 19.7 Gy lower and the median D_max_ was 24 Gy lower with VMAT (*P* < 0.001 for both). Significantly lower doses were also found with VMAT for the contralateral cochlea, with D_mean_ and D_max_ being lower by 9.5 and 11.2 Gy, respectively.

**Table 3:** Organs at risk for the 90-patient cohort

	Median (Range)		
	IMRT	VMAT	P*
Brain^a^			
D_mean_ (Gy)	26.7 (12.0–36.5)	27.6 (15.8–36.0)	0.41
D_max_ (Gy)	63.8 (62.6–66.2)	63.5 (62.0–64.9)	0.07
V_30Gy_ (%)	34.5 (11.4–58.0)	38.0 (19.3–61.5)	0.16
V_60Gy_ (%)	3.7 (0.7–9.6)	3.3 (1.1–12.1)	0.62
Brain^b^			
GI_50Gy_	1.9 (1.5–2.3)	1.9 (1.5–2.4)	0.08
GI_60Gy_	5.0 (2.8–31.6)	6.4 (2.3–13.2)	0.05
Brainstem			
D_mean_ (Gy)	28.7 (7.8–46.3)	19.3 (0.8–39.2)	<0.001
D_max_ (Gy)	53.7 (18.2–61.6)	53.2 (2.2–60.4)	0.13
V_30Gy_ (%)	39.2 (0.0–88.1)	26.1 (0.0–71.4)	0.005
Ipsilateral Hippocampus			
D_mean_ (Gy)	53.0 (16.0–62.5)	46.8 (1.6–62.3)	0.06
D_max_ (Gy)	60.5 (24.2–64.0)	55.7 (3.2–63.5)	0.12
D_100%_ (Gy)	31.7 (3.0–59.1)	19.7 (1.0–59.6)	0.03
Contralateral Hippocampus			
D_mean_ (Gy)	20.3 (10.9–55.9)	22.5 (0.9–55.8)	0.25
D_max_ (Gy)	34.7 (18.3–61.9)	39.3 (1.7–62.8)	0.26
D_100%_ (Gy)	13.1 (2.1–46.3)	13.4 (0.7–50.8)	0.88
Bilateral Hippocampus			
D_mean_ (Gy)	35.7 (13.7–57.0)	35.1 (1.3–56.3)	0.79
D_max_ (Gy)	60.5 (24.2–64.0)	56.0 (3.2–63.5)	0.15
D_100%_ (Gy)	12.3 (2.1–46.3)	11.6 (0.7–47.0)	0.95
Optic Chiasm			
D_mean_ (Gy)	35.8 (1.3–52.9)	35.7 (1.5–52.5)	0.84
D_max_ (Gy)	51.6 (2.3–55.7)	51.3 (1.8–53.9)	0.52
Ipsilateral Optic Nerve			
D_mean_ (Gy)	20.9 (0.7–49.7)	20.7 (0.9–47.8)	0.73
D_max_ (Gy)	35.9 (1.1–55.7)	43.6 (1.3–54.5)	0.72
Contralateral Optic Nerve			
D_mean_ (Gy)	9.9 (0.6–46.4)	12.2 (0.8–44.9)	0.46
D_max_ (Gy)	19.5 (1.0–55.0)	23.6 (1.2–54.7)	0.80
Ipsilateral Cochlea			
D_mean_ (Gy)	25.6 (3.0–61.9)	5.9 (0.5–48.2)	<0.001
D_max_ (Gy)	30.8 (3.6–62.3)	6.8 (0.6–53.7)	<0.001
Contralateral Cochlea			
D_mean_ (Gy)	12.7 (1.7–25.8)	3.2 (0.4–20.0)	<0.001
D_max_ (Gy)	15.0 (2.0–29.6)	3.8 (0.4–23.7)	<0.001
Ipsilateral Eye			
D_mean_ (Gy)	3.6 (0.4–24.4)	5.1 (0.6–22.5)	0.08
D_max_ (Gy)	7.9 (1.0–49.6)	15.1 (1.0–46.5)	0.32
Contralateral Eye			
D_mean_ (Gy)	3.1 (0.4–8.5)	4.0 (0.6–20.9)	0.08
D_max_ (Gy)	7.1 (0.5–40.2)	9.9 (1.0–37.2)	0.07
Ipsilateral Lens			
D_mean_ (Gy)	2.2 (0.4–5.6)	2.7 (0.5–14.4)	0.18
D_max_ (Gy)	2.6 (0.4–8.3)	3.1 (0.6–16.8)	0.41
Contralateral Lens			
D_mean_ (Gy)	1.7 (0.3–19.7)	2.4 (0.5–14.3)	0.02
D_max_ (Gy)	2.0 (0.3–31.2)	2.7 (0.6–17.1)	0.05

*IMRT* intensity modulated radiation therapy, *VMAT* volumetric arc therapy, *D*_*min*_ minimum dose, *D*_*mean*_ mean dose, *D*_*max*_ maximum dose, *D100%* maximum dose covering 100% of organ, *V*_*XXGy*_ volume receiving XX Gy or more, *GI* gradient index

*From Wilcoxon-Mann–Whitney rank sum analysis ^a^Brain organ at risk volume excludes the gross tumor volume, brainstem and optic chiasm volumes ^b^Brain volume does not exclude the gross tumor volume, brainstem and optic chiasm volumes

A separate analysis of the PTV and select OARs was performed for the 75 patients treated on linear accelerators equipped with high definition multileaf collimators. Similar to the full patient cohort, the mean PTV doses were significantly different, with D_mean_ for the PTV being a median value of 57.1 Gy for IMRT and 56.4 Gy for VMAT (*P* < 0.001). Similarly, the median values of D_mean_ for the brainstem were 29.2 Gy for IMRT and 18.6 Gy for VMAT (*P* < 0.001). For the ipsilateral cochlea, the median values of D_mean_ were 27.4 Gy for IMRT and 5.7 Gy for VMAT (*P* < 0.001), while for the contralateral cochlea they were 13.1 Gy for IMRT and 3.1 Gy for VMAT (*P* < 0.001). The ipsilateral hippocampal D_100%_ was 32.8 Gy for IMRT and 18.8 Gy for VMAT (*P* = 0.02).

**Fig. 1:**
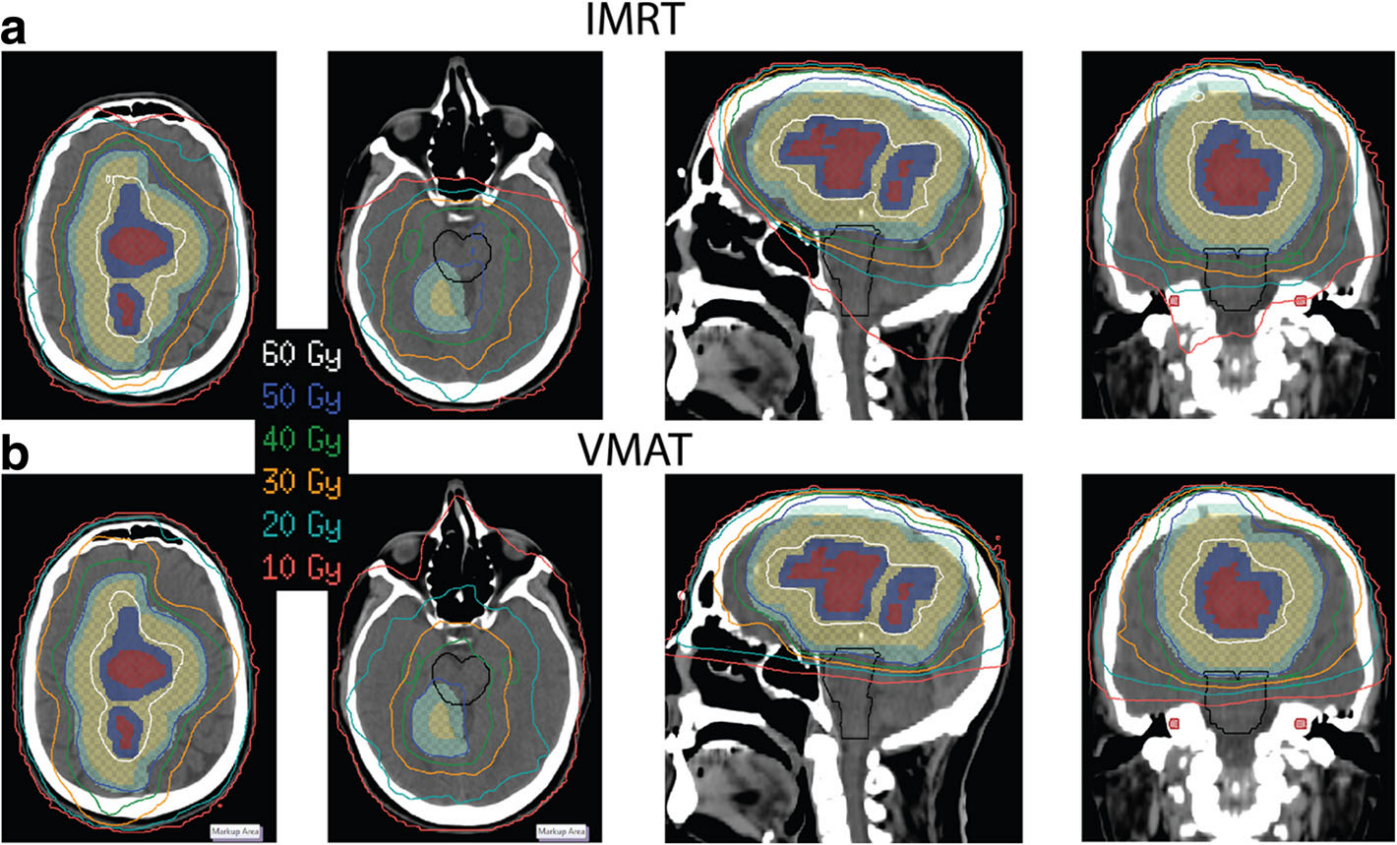
Isodose lines for one VMAT patient on two representative axial views as well as sagittal and coronal views. **a** IMRT plan **b** VMAT plan. The target planning volumes are in colorwash: GTV (*maroon*), bPTV (*dark blue*), CTV (*gold*), PTV (*aquamarine*). The brainstem is outlined in *black*, the cochleae in *red* colorwash, and the hippocampi in *green*

Selected doses and volumes for the six patients included in the VMAT cohort who had additional clinical IMRT plans are shown in Tables 4 and 5. Isodose lines for one patient are shown in Fig. 1. As with the larger cohort, the VMAT plans provide similar target coverage and dose homogeneity within the PTV and bPTV, and similar doses and dose gradients within the brain. The target volume coverage for both the IMRT and VMAT plans were considered clinically acceptable (≥95%), with only small differences in dose to the brain. With respect to the optic chiasm and optic nerves, D_max_ was < 54 Gy for all but one IMRT plan, with the VMAT plans being neither consistently higher nor lower than the IMRT plans. Both D_mean_ and D_max_ for the eyes were well below specified constraints for all plans. The ipsilateral hippocampal D_100%_ was consistently lower for the VMAT plans, and the contralateral and bilateral D_100%_ were lower for 4 of 6 plans. For the brainstem and ipsilateral cochlea, D_mean_ was consistently lower with VMAT, and D_mean_ for the contralateral cochlea was either equally small or lower for the VMAT plans. Fig. 1 shows the IMRT and VMAT plans for one patient, with similar bPTV and PTV coverage in both. The 40-Gy isodose line covers the brainstem in the central image for the IMRT plan, but only partially covers the brainstem with VMAT. Finally, the cochleae are partially or completely covered by the 10 Gy isodose line with IMRT, but with VMAT all doses on the slice containing the cochleae are < 10 Gy.

**Table 4:** Planning target volumes for 6 patients in the 45-patient VMAT cohort with both IMRT and VMAT plans

	Median (Range)		
	IMRT	VMAT	P*
bPTV			
D_min_ (Gy)	58.9 (55.6–59.7)	58.8 (57.2–60.0)	0.12
D_mean_ (Gy)	62.0 (61.7–62.3)	62.1 (61.9–62.6)	0.83
D_max_ (Gy)	63.9 (63.2–65.8)	63.7 (63.1–64.7)	0.35
V_60Gy_ (%)	99.8 (97.8–100.0)	99.7 (98.7–100.0)	0.17
II (Gy)	2.0 (1.3–2.4)	1.8 (1.1–2.4)	0.35
PTV			
D_min_(Gy)	45.4 (40.8–47.9)	41.2 (35.2–45.4)	0.03
D_mean_ (Gy)	56.7 (55.7–58.0)	56.1 (54.7–57.8)	0.03
D_max_ (Gy)	63.9 (63.2–65.8)	63.7 (63.1–64.7)	0.35
V_50Gy_ (%)	98.5 (95.8–99.8)	96.7 (95.9–98.9)	0.12
II (Gy)	11.4 (10.9–12.0)	11.8 (11.6–12.5)	0.17

*IMRT* intensity modulated radiation therapy, *VMAT* volumetric arc therapy, *bPTV* boost planning target volume prescribed to 60 Gy, *PTV* planning target volume prescribed to 50 Gy, *D*_*min*_ minimum dose, *D*_*mean*_ mean dose, *D*_*max*_ maximum dose, *V*_*XXGy*_ volume receiving XX Gy or more, *II* inhomogeneity index

*From Wilcoxon signed rank sum analysis

**Table 5:** Organs at risk for 6 patients in the 45-patient VMAT cohort with both IMRT and VMAT plans

	Median (Range)		
	IMRT	VMAT	P*
Brain^a^			
D_mean_ (Gy)	27.3 (25.8–38.9)	26.1 (25.1–35.6)	0.03
D_max_ (Gy)	63.6 (63.1–65.8)	63.5 (63.1–64.7)	0.75
V_30Gy_ (%)	36.6 (32.2–61.1)	34.8 (32.0–61.5)	0.60
V_60Gy_ (%)	4.7 (2.4–7.4)	4.0 (1.8–6.9)	0.03
Brain^b^			
GI_50Gy_	1.8 (1.7–2.0)	1.9 (1.7–2.1)	0.12
GI_60Gy_	6.3 (3.3–8.2)	7.2 (3.2–8.8)	0.05
Brainstem			
D_mean_ (Gy)	24.3 (16.9–33.8)	14.1 (9.5–27.2)	0.03
D_max_ (Gy)	53.8 (51.2–59.4)	53.5 (49.5–59.9)	0.12
V_30Gy_ (%)	27.0 (15.3–56.2)	19.3 (10.2–45.0)	0.03
Ipsilateral Hippocampus			
D_mean_ (Gy)	45.5 (33.8–58.9)	41.7 (35.4–58.7)	0.07
D_max_ (Gy)	56.2 (50.6–63.0)	56.5 (51.4–63.3)	0.25
D_100%_ (Gy)	26.5 (12.1–52.8)	10.0 (8.4–51.1)	0.03
Contralateral Hippocampus			
D_mean_ (Gy)	21.0 (15.8–45.4)	23.7 (15.1–42.0)	0.25
D_max_ (Gy)	44.6 (32.8–62.2)	44.5 (31.7–62.2)	0.12
D_100%_ (Gy)	14.4 (2.5–26.0)	9.8 (3.9–20.8)	0.25
Bilateral Hippocampus			
D_mean_ (Gy)	35.9 (24.6–45.8)	33.7 (25.0–42.1)	0.25
D_max_ (Gy)	56.6 (50.6–63.0)	56.5 (51.4–63.3)	0.35
D_100%_ (Gy)	14.4 (2.5–26.0)	9.8 (3.9–20.8)	0.25
Optic Chiasm			
D_mean_ (Gy)	37.2 (19.6–50.5)	36.9 (19.9–52.3)	0.35
D_max_ (Gy)	50.0 (30.7–55.7)	52.0 (25.0–53.9)	0.35
Ipsilateral Optic Nerve			
D_mean_ (Gy)	11.7 (4.5–39.9)	18.9 (7.8–38.1)	0.35
D_max_ (Gy)	33.6 (14.2–55.1)	34.1 (15.4–53.9)	0.60
Contralateral Optic Nerve			
D_mean_ (Gy)	9.3 (6.7–29.6)	13.8 (7.5–31.0)	0.05
D_max_ (Gy)	23.5 (10.3–54.5)	25.9 (12.1–52.7)	0.92
Ipsilateral Cochlea			
D_mean_ (Gy)	19.3 (6.2–26.3)	2.1 (1.4–5.8)	0.03
D_max_ (Gy)	21.0 (8.4–28.6)	2.4 (1.5–10.3)	0.03
Contralateral Cochlea			
D_mean_ (Gy)	8.9 (1.4–23.5)	1.9 (1.4–2.3)	0.03
D_max_ (Gy)	10.2 (1.5–24.2)	2.3 (1.5–3.0)	0.03
Ipsilateral Eye			
D_mean_ (Gy)	3.5 (0.8–11.8)	6.1 (2.8–10.9)	0.25
D_max_ (Gy)	13.5 (1.5–50.6)	15.7 (4.7–34.7)	0.92
Contralateral Eye			
D_mean_ (Gy)	4.7 (1.6–5.6)	4.9 (3.3–8.6)	0.17
D_max_ (Gy)	8.6 (2.6–41.5)	11.0 (6.4–34.1)	0.60
Ipsilateral Lens			
D_mean_ (Gy)	2.4 (0.5–6.0)	2.8 (1.9–4.0)	0.75
D_max_ (Gy)	2.8 (0.6–7.8)	3.3 (2.3–4.9)	0.92
Contralateral Lens			
D_mean_ (Gy)	2.3 (1.1–3.6)	2.6 (2.0–3.9)	0.35
D_max_ (Gy)	3.4 (1.3–3.9)	2.9 (2.2–4.8)	0.75

*IMRT* intensity modulated radiation therapy, *VMAT* volumetric arc therapy, *D*_*min*_ minimum dose, *D*_*mean*_ mean dose, *D*_*max*_ maximum dose, *D100%* maximum dose covering 100% of organ, *V*_*XXGy*_ volume receiving XX Gy or more, *GI* gradient index

*From Wilcoxon signed rank sum analysis ^a^Brain organ at risk volume excludes the gross tumor volume, brainstem and optic chiasm volumes ^b^Brain volume does not exclude the gross tumor volume, brainstem and optic chiasm volumes

## Discussion

Dose to the planning target volumes shown in Table 2 demonstrate the non-inferiority of VMAT for the tumor coverage in this patient cohort, where ≥ 94.7% coverage was achieved for each PTV and bPTV planned with VMAT. These findings did not change when patients planned with conventional multileaf collimators were omitted from the analysis. The small differences in target volume coverage and dose homogeneity are unlikely to be clinically significant, as exemplified by the six patients with both IMRT and VMAT plans in Table 4. For all patients, while the dose to the brain and optic nerves was not substantially different, the dose to the brainstem and cochleae was significantly improved with VMAT. This finding is demonstrated in Fig. 1, which shows that the IMRT dose distribution exceeds 10 Gy several cm inferior to the PTV. This dose distribution occurs because noncoplanar beams are used for IMRT treatment planning, but are only rarely needed with VMAT. Thus, VMAT planning leads to superior sparing of the brainstem and cochleae without sacrificing PTV coverage.

Reducing radiation dose to the normal tissues outside the PTV is important, particularly for patients who will likely have disease recurrence and may require further radiotherapy. In order to avoid sensorineural hearing loss, which may occur as early as 3 months after completing RT, the Quantitative Analyses of Normal Tissue Effects in the Clinic (QUANTEC) group recommended a mean cochlear dose of ≤ 35–45 Gy [32]. Additionally, because a threshold for hearing loss could not be determined, the study group recommended keeping the dose as low as possible. A D_mean_ ≤ 35 Gy was achieved for all contralateral cochleae, 34 ipsilateral cochleae in the IMRT cohort and 42 in the VMAT cohort. This is in keeping with the dose constraints used during treatment planning. However, considering that the mean ipsilateral and contralateral cochlear doses are lower by 19.7 and 9.4 Gy with VMAT, respectively, hearing could be preserved even with further irradiation. This was further exemplified by the six patients with IMRT and VMAT plans, where D_mean_ was reduced by 4.7–23.3 Gy with VMAT. While QUANTEC recommendations for the brainstem did not provide definitive dose-volume constraints [33], again minimizing dose to this critical structure could be important for subsequent irradiation. For the six patients with both IMRT and VMAT plans, the brainstem D_mean_ was reduced by 4.5–15.3 Gy. The impact of normal tissue sparing on treatment toxicity merits further study.

Previous planning studies, in which IMRT and VMAT plans were created for small cohorts of 10–14 patients [20, 24, 25], showed good PTV coverage for both IMRT and VMAT and similar sparing of reported critical structures with the exception of the contralateral optic apparatus. For the contralateral optic nerve, retina, anterior globe, and lens, VMAT was found to be superior [24, 25]. In the present study of 90 patients, the globe and retina were contoured as a single structure, and no statistically significant difference in dose to the contralateral eye or optic nerve was observed. Previous studies [24, 25] showed the mean brainstem D_mean_ to be 28.8–33.4 Gy for IMRT and VMAT plans. The ipsilateral mean cochlear D_mean_ were reported to be 35.8–53.9 Gy and the contralateral mean cochlear D_mean_ were reported to be 10.7–12.7 Gy [25]. In one study [25], higher brainstem D_mean_ were observed for non-coplanar IMRT, while higher ipsilateral cochlear D_mean_ were seen for coplanar IMRT. Differences in brainstem [24, 25] and cochlear doses [25] were not found to be significant. Direct comparison with these two previous studies is difficult because the abovementioned studies employed a 60-Gy dose to the entire PTV, while in the present study the PTV was prescribed to 50 Gy the boost PTV to 60 Gy. The values of the brainstem and cochlear D_mean_ for the IMRT plans for the entire 90-patient cohort in the present study (Table 3) are line with the previous studies, and the significantly improved sparing with VMAT was not observed in the earlier studies. To the best of our knowledge, this work is the first study that demonstrates the superiority of sparing of the brainstem and cochleae with VMAT compared with IMRT.

Radiation to the hippocampus has been associated with neurocognitive impairment in patients undergoing cranial irradiation [34]. The possibility of using hippocampal sparing to improve quality of life has been the focus of study for patients with limited expected survival, including those receiving whole brain irradiation for intracranial metastases [35–37]. A study of 18 adult patients with brain tumors suggested a D_40%_ > 7.3 Gy is associated with cognitive impairment [34], while a separate study also of 18 patients suggested a relationship between the left hippocampal D_max_ and a decline in learning and recall [38]. The RTOG 0933 study on hippocampal avoidance during whole brain irradiation limited the D_100%_ to ≤ 9 Gy and D_max_ to ≤ 16 Gy in 3 Gy fractions [36], which would be equivalent to a D_100%_ of ≤ 11.25 Gy and D_max_ of ≤ 20 Gy in 2 Gy fractions if an α/β of 2 is assumed. A published case study pointed to the possibility of using VMAT to achieve hippocampal sparing for low-grade gliomas [39]. In the present study, the hippocampi were not treated as avoidance structures during treatment planning. The median ipsilateral D_100%_ was lower by 12 Gy for the VMAT cohort (*P* = 0.03), and this is supported by the six patients with both VMAT and IMRT plans, with increased hippocampal sparing of 1.6–17.3 Gy. None of the investigated hippocampal doses were found to be statistically significant, and both the contralateral and bilateral D_100%_ and D_max_ were similar between the VMAT and IMRT cohorts. These results suggest a trend towards VMAT resulting in improved hippocampal sparing, which may offer a distinct advantage for certain patients. Using the hippocampi as avoidance structures may result in more optimal dose distributions. Avoidance should be considered with regard to current ASTRO guidelines, which do not recommend compromising target coverage to increase hippocampal sparing [2]. The current results are promising, however, and may encourage clinical investigation of this topic.

The observation of a shorter treatment time for VMAT is supported by previous studies [20, 24, 25]. In the present study, more monitor units were required for VMAT (453 vs. 347 for IMRT), but this number is consistent with the aforementioned studies that show mean values of 321–495 monitor units. Time is saved because of the limited number of beams used in VMAT as well as the coplanar configuration of the beams, obviating the need to enter the treatment vault between treatment fields. This may improve clinical efficiency, and allow more patients to be treated in a shorter amount of time. A shorter treatment time may also improve the treatment experience of patients who have difficulty tolerating a treatment mask.

One of the differences between the present study and previous published comparative analyses of IMRT versus VMAT is that the latter were planning studies, whereas all data from this investigation are from clinically delivered plans. Consequently, dose statistics were evaluated from different patients within the VMAT and IMRT cohorts. However, statistical analysis of the patient characteristics, including tumor location and PTV volume (Table 1), suggests that the patient populations are very similar. Thus, it is unlikely that differences in the sparing of normal tissues are solely due to patient inhomogeneity. This is further supported by analysis of six patients who had comparative VMAT and IMRT plans developed before the decision was made to proceed with the respective VMAT plans (Tables 4 and 5). Finally, using clinical plans without population inhomogeneity removes the possible bias that can occur from a comparison (or dual)-planning study. Planning studies with limited patient cohorts are important to introduce new modalities or techniques. However, such studies often leave open questions as to whether the new modality behaves as promised when applied to the clinic or if the institution publishing the study actually uses this method. This report of our clinical experience adds support to previous planning studies and further shows that superior brainstem and cochlear sparing can be achieved with VMAT. Our institution has more than ten years of experience with IMRT planning for GBMs, and the present result demonstrating the superiority of VMAT was unexpected. While further planning studies could have been done to further optimize the IMRT plans, this did not appear to be warranted in light of the other advantages of VMAT, including shorter treatment time. It is hoped that other institutions will also find this modality advantageous in the treatment of GBMs.

## Conclusions

In our analysis of 90 patients treated with a simultaneous integrated boost technique for GBM, we have found that superior sparing of the brainstem and cochleae can be achieved with VMAT without sacrificing target coverage or sparing of other organs at risk. Hippocampal sparing appears to be improved for certain patients. Treatment time can also be reduced by a median value of five minutes per patient when VMAT is used, which may improve clinical efficiency as well the treatment experience of patients who have difficulty tolerating the treatment mask. Use of VMAT for the treatment of GBM patients thus appears to offer no inferiority and distinct advantages when compared with IMRT. While normal tissue sparing was not universally improved for all organs at risk, cochlear and hippocampal sparing could enhance the quality of life for these patients with limited expected survival. The clinical impact of the sparing of normal tissues merits further study but may be especially important for these GBM patients who will likely have tumor recurrence and may require re-irradiation.
